# Analysis of factors affecting discharge with the personal consent of hospitalized patients: A cross‐sectional study

**DOI:** 10.1002/hsr2.1447

**Published:** 2023-08-01

**Authors:** Zahra Babaei, Masoumeh Alizadeh, Soodeh Shahsawari, Azar Jihoni‐Kalhori, Ramin Cheraghbeigi, Rahman Sotoudeh, Ali Mohammadi

**Affiliations:** ^1^ Department of Health Information Management Mohamad Kermanshahi Hospital, Kermanshah University of Medical Sciences Kermanshah Iran; ^2^ Department of Health Information Management Farabi Hospital, Kermanshah University of Medical Sciences Kermanshah Iran; ^3^ Department of Health Information Technology, Faculty of Paramedical Sciences Kermanshah University of Medical Sciences Kermanshah Iran; ^4^ Strategic Purchase for Clinical Management Social Security Organization Kermanshah Iran; ^5^ Department of Administrative Affairs Shohada Hospital, Social Security Organization Kermanshah Iran

**Keywords:** consent, discharge, hospitalization, patient

## Abstract

**Background and Aims:**

Discharge by personal satisfaction is a condition in which the patient leaves the hospital before completing the care period against medical advice. Thus, this study aims to identify and analyze the reasons for discharge with the personal satisfaction of hospitalized patients.

**Methods:**

The study was descriptive‐analytical being performed in 2021. The study population was 2869 discharged inpatients with personal satisfaction. Sampling was done by random and census. The data were collected using a checklist and a researcher‐made questionnaire whose validity and reliability were confirmed. The data were analyzed using SPSS24 by K‐Score test for qualitative and variance for quantitative variables.

**Results:**

The discharge rate by personal satisfaction was 7.01%, the average age was 42 years, and the average length of stay was 4 days. Further, 57.1% of patients were female, 63.7% were married, and 21% were babies. A total of 22.77% of the patients were discharged with the father's consent, of which 13.2% were re‐admitted. The most common reasons for the discharge were feeling of recovery (47.2%), the hospital being educational (30%), and dissatisfaction with the services of doctors (51.6%). Discharge with personal satisfaction had a significant relationship with the length of hospitalization (*p* < 0.001) and type of hospital (*p* = 0.04).

**Conclusion:**

The feeling of recovery, the educational nature of the hospital, and dissatisfaction with the services of doctors were the most common reasons for discharge with personal satisfaction. Therefore, monitoring the provision of services, establishing proper patient‐doctor communication, and increasing the awareness of patients and parents could reduce this type of discharge and its consequences.

## 1 INTRODUCTION

Nowadays, retaining customers in the healthcare system has become more difficult to the past since the increased number of organizations providing medical services, on the one hand, and the increased awareness of medical issues and quality care, on the other hand, offer applicants a wider range of choices. Therefore, in the competition arena, an organization will be more successful if it makes more efforts to satisfy its customers.^1^ The health system aims to produce health by providing services to patients. For this reason, if there is no trust in the service provider organizations, the health sector will lose its identity. Indeed, patient satisfaction with medical care is a measure of his/her understanding of the care quality.^2^

Satisfaction is the basis for choosing a healthcare facility in the future by the patient or recommending it to other patients. Patient dissatisfaction also leads to poor care cooperation, causing a waste of resources and decreasing the quality of care. Therefore, trying to obtain patient satisfaction should be one of the important goals of healthcare organizations. One of the problems that may arise due to patient dissatisfaction with the services received is discharge with personal consent, indicating the existence of a significant issue.^3^ This ultimately causes the stagnation of work and the poor financial condition of the centers, irreparably damaging them ^4^ with increased costs and decreased income.^5^

Self‐satisfied discharge refers to a condition in which the patient is discharged before completing the course of care against medical advice.^2^, ^5^, ^6^ There is great variation in the discharge rate by personal consent from one country to another. Approximately 1%–2% of all hospital discharges in the United States are personal.^7^ Also, this rate has been reported as 4% in Saudi Arabia, 0.72% in Nigeria, and 10.3% in Iran.^8^ In addition to the problems it creates for the hospital, the type of discharge is also an important issue for the patient and healthcare providers^8^ because it leads to adverse health outcomes, rehospitalization, illness, and death.^7^, ^8^, ^9^, ^10^

According to research, the rate of rehospitalization in the first 2 weeks after discharge in patients with personal consent is higher than in those discharged with a doctor's order.^2^, ^9^ The rehospitalization rate in such patients increases since their condition at home is worsened, or the therapeutic process is disrupted.^11^ This rate in Canada is 10% ^1^ and, in the United States, it is between 0.8% and 2.2%,^12^ while in Iran, it is 3% in people with a mental health condition and 20% in the emergency department.^11^

The goal of a health system is to ensure the health of people in a society. To achieve this, it must provide desirable services; the desirability of such services is evaluated by monitoring and controlling them. One method to assess health services is to investigate the level of satisfaction of patients. The reason for the great tendency of health researchers to investigate and analyze the factors affecting patient satisfaction is determined according to these points and the number of studies in this field in different countries and years. These studies in different volumes and designs had variable results; however, their common result, patient satisfaction with the discharge, has the opposite relationship with personal desire.^13^

Malekzadeh et al.,^6^ in a cross‐sectional descriptive study, investigated the status of discharge by personal consent. Based on their results, the most common reasons for such discharge were relative recovery, lack of trust in the quality of hospital services, and emotional motives.^6^ Ghorbinia and Moradi^5^ descriptively analyzed the causes of discharge by personal consent. In their study, the discharge rate was 8.69%, the most common reason was the feeling of recovery, and there was a statistical relationship between such discharge with marital status and type of insurance. Ashrafi et al.^11^ investigated discharge with personal satisfaction, besides its causes and predictive factors. In this research, the most common cause of the discharge was personal problems and going to other centers. Based on this study, to reduce the rate of discharge with the satisfaction of the person, it is required to create a suitable environment, provide appropriate services, and meet the needs of patients and companions.^11^

Self‐directed discharge is a multi‐dimensional category and includes several factors.^2^ Identifying these factors is very important to recognize those more likely to have this type of discharge. Accordingly, it is possible to inform people about its consequences, which prevents this process, thus reducing the rate of rehospitalization and mortality. Therefore, this study aimed to identify and analyze the causes of discharge with personal satisfaction in hospitalized patients.

## 2 METHODS

The study was descriptive‐analytical and done cross‐sectionally among patients (in the inpatient departments of selected hospitals, including Imam Reza (a.s.) and Motazadi from the University of Medical Sciences and Shohada and Hazrat Masoumeh (a.s.) from the Social Security Organization) discharged with personal consent in Kermanshah city (west of Iran) in 2021. Two general and two neonatal hospitals from two different organizations were selected to compare the hospitals and their patients.

The study population consisted of 2869 patients discharged with personal consent (Imam Reza 2766, Motazadi 22, Shohada 15, and Hazrat Masoumeh 66). In Imam Reza (a.s.) hospital, simple random sampling was used due to high statistics, while, in other hospitals, the census method was applied due to low statistics. The data were collected using a checklist and a researcher‐made questionnaire. The validity of the checklist and questionnaire was confirmed by the opinion of 10 experts (three information management specialists, four nurses, and three physicians), and the reliability of the questionnaire was confirmed by Cronbach's alpha of 85%.

The checklist included five sections of patient information, disease, hospital and doctor, insurance, and para‐clinical services. Data were collected by preparing a list of discharged patients with personal consent, extracting their records from the hospital information system, and studying the records. A questionnaire was used to collect data on the reasons for self‐directed discharge, which consisted of two parts: the first included satisfactory characteristics, patient demographic information, and admission and discharge times, and the second included reasons related to the patient (10 reasons), hospital (6 reasons), and service providers (7 reasons).

To collect the data for this section, the phone/mobile number of the patient and companion were retrieved from the system; then, the patient was contacted first, and by introducing him/herself, the reason for discharge was asked with personal consent. If they did not answer or did not know because they were elderly, children, or disabled, the companion was asked. Data were analyzed using (SPSS24 software), descriptive statistics, mean, standard deviation, and analysis of variance. The significance level was considered at 0.05.

Ethical considerations were observed in data collection. Patient and service provider identification information was not collected. In the phone call with the patient or companion, if they did not want to answer or give a reason, there was no compulsion to do so. In the case of all patients, personal consent to provide information was obtained. Finally, this project was approved by the Ethics Committee of Kermanshah University of Medical Sciences under the code KUMS.REC.1396.128.

## 3 RESULTS

In this study, discharge with personal consent was investigated in four hospitals (two general and two neonatal hospitals) related to the University of Medical Sciences and Social Security in Kermanshah. The research findings showed that the total discharge rate with personal satisfaction was 7.01%, 8.86% for the University of Medical Sciences, 0.86% for social security, 10.66% for general, and 0.6% for newborns. The discharge rate with personal consent in the University of Medical Sciences general hospital was 10.98%, while it was 1.66% for social security. Also, the rate of discharge with the personal consent of newborns in the hospital of the University of Medical Sciences was 0.35%, while it was 0.77% for social security.

The average age of the patients was 42 years, and the average length of stay was 4 days. The largest number of patients, with 57.1%, were women. Further, 63.7% were married, and 21% were babies. Additionally, 28.1% had less education than a diploma, 21.8% had a diploma, and 17.5% were illiterate, while people with higher education, i.e., master's degree and above, were 1.7%. Housewives, with 41.3%, were the most, and students, with 3%, were the least discharged patients with personal consent. Also, 86.5% had no underlying disease.

A total of 22.77% of the patients were discharged with the father's consent, followed by the son and the wife (20.79%). Most of these patients were admitted on Saturday (16.8%) and fewer on Thursday (11.9%); also, 42.6% were admitted in the morning, and 59.4% were discharged in the morning. Additionally, 13.2% of patients discharged by personal consent were re‐admitted.

In this study, the reasons for discharge with personal satisfaction were investigated for three cases related to service providers (personnel and doctors), patients, and hospitals. For personal reasons related to the patient, feeling better (47.2%) was the most common, and being a traveler and not having a companion were the least reasons (1.2%) (Tables 1 and 2).

**Table 1:** Frequency of personal discharge reasons related to the patient.

Reasons related to the patient	Frequency	Percent
Passenger	3	1.2
Financial problems	7	2.8
Not having a companion	3	1.2
Doctor's suggestion	18	7.1
Feeling better	120	47.2
Family matters	15	5.9
Transfer to other medical centers	36	14.2
Not native	10	3.9
Fatigue of the patient from a long hospitalization	25	9.8
Having children or relatives responsible for them	17	5.6
Total	254	100

**Table 2:** Frequency of personal reasons for discharge by hospital.

Personal reasons	Hospital							
	Infants				General			
	University		Social security		Social security		University	
	Frequency	Percent	Frequency	Percent	Frequency	Percent	Frequency	Percent
Passenger	0	0	0	0	0	0	3	100
Financial problems	1	14.3	0	0	0	0	6	85.7
Not having a companion	0	0	0	0	1	33.3	2	66.7
Doctor's suggestion	0	0	0	0	0	0	18	100
Feeling better	8	6.7	44	36.7	5	4.2	63	52.5
Family matters	0	7	6	40	4	26.7	5	33.3
Transfer to other medical centers	0	7	8	22.2	2	5.6	26	72.2
Not native	4	40	0	0	0	0	6	60
Fatigue of the patient from a long hospitalization	4	16	0	0	1	4	20	80
Having children or relatives responsible for them	3	17.6	8	47.1	2	11.1	4	23.5
Exact test	*p* = 0.04							

For factors related to the hospital, being educational was the highest reason, with 30% (Table 3). For reasons related to healthcare providers, lack of satisfaction with doctor services (51.6%) and nursing services (18.8%) were the highest, while disrespect to the patient or companion (1.6%) was the lowest reason (Table 4).

**Table 3:** Frequency of **hospital**‐related personal discharge reasons.

Hospital reasons	Frequency	Percent
The oldness of the hospital	1	5
The educational nature of the hospital	6	30
Hospital facilities	2	10
The hospital dirtiness	5	25
Overcrowding and high density of patients	3	15
Lack of para‐clinical facilities and equipment	3	15
Total	20	100

**Table 4:** Frequency of personal discharge reasons related to **healthcare providers**.

Staff/physician reasons	Frequency	Percent
Dissatisfaction with medical services	33	51.6
Dissatisfaction with nursing services	12	18.8
Delay in patient care and treatment	8	12.5
Disrespect to the patient and companion	1	1.6
Not giving information about the patient's condition	3	4.7
Lack of patient visits by specialists	5	7.8
Not paying attention to the pain treatment in the patient	2	3.1
Total	64	100

## 4 DISCUSSION

Nowadays, with information technologies, awareness of healthcare facilities and their quality has increased, making it possible to be compared. The number of patients who leave the hospital with personal satisfaction indicates issues that need attention. In addition, discharge with personal consent leads to increased costs, loss of working days, disability, or even death for patients.^14^ Therefore, in today's competitive world, to attract and increase customers, it is necessary for hospitals to continuously investigate the reasons for discharge by personal consent and complaints, thus solving problems and improving the conditions in the future.

The overall rate of discharge by personal satisfaction was 7.01. It has been reported between 0.8% and 2.2% in American hospitals, while it is 1.8%, 1.1%, and 4.2% in England, Canada, and Nigeria, respectively. In Iran, this rate is between 3% in Psychiatric hospitals, varying up to 20% in emergency departments.^15^ The discharge rate by personal consent in hospitalized patients of Kashan University of Medical Sciences is 10.3%, Tehran University of Medical Sciences is 7.17%, and Boali Children's Hospital in Sari is 22%.^16^

In this study, the average age of the patients was 42 years, and the average length of stay was 4 days. The largest number of patients (57.1%) were women, 63.7% were married, and 21% were babies. Ghorbania et al.^5^ showed that the average age of patients discharged by personal consent was 33.76, 58.6% were women, 66.3% were married, and their average length of stay was 5 days.^5^ In the study by Mohammadi Kejidi et al.,^14^ the average age of hospitalized patients discharged with personal consent was 48.72 years, 60.9% were male, 81.3% were married, and the average length of their stay was 3 days. Also, Olasinde et al.^17^ stated that the overall rate of discharge by personal consent was 4.1%, 53.5% of the patients were women; because this study was conducted on people under 18 years of age, 79% were children, with 72.1% discharged with the father's consent. In Motazedi et al.,^18^ the overall rate of discharge by personal consent was 3.8%, the average age was 45 years, the average length of stay was 5 days, 56% were male, and 79.5% were married. According to this study and others, most of the people were young and more prone to discharge with personal satisfaction due to being employed, the head, and the breadwinner of the household; hence, after partial recovery and due to the lack of financial means, they wanted to be discharged.

A total of 28.1% of patients discharged with personal consent had no high school diploma. Housewives, with 41.3%, were the most discharged patients with personal consent. Also, 86.5% of these patients had no underlying disease. In a study by Setareh et al.,^19^ 83.2% of the patients had no high school diploma, and 66.6% had no job. In Motazedi et al.,^18^ 61.8% of patients had no high school diploma. Also, in Najiba Rasool^16^ 58.1% of patients were housewives, and the least were employees, with 3.1%. In this study, 22.77% of the discharges were with the father's consent, and 13.2% of the patients discharged with personal consent were re‐admitted, while in the study of Ravanshad et al., the most consent for discharge was given by the father, and 17.7% were re‐hospitalized. They said that it was because the treatment process was not completed.

Considering that the study is related to children and the discharge was done with the father's consent, full justification of the parents is necessary to make the right decision.^15^ In Kamaluddin's study, 15%^20^ and, in Asadi's study, 9.06% of patients were re‐admitted 15 days after discharge with personal consent.^21^ Two hospitals in this study were related to children, the age and legal condition of whom were such that they were allowed to be treated and discharged by their parents; thus, about 30% were discharged with the father's consent. Therefore, it is necessary to give training and counseling to parents about the consequences of personal consent discharge.

Reasons for discharge by personal consent were investigated for three cases related to healthcare providers, patients, and hospitals. For patient‐related reasons, the feeling of recovery was the most common (47.2%). The feeling of getting better caused the discharge of 44.2% of men and 55.8% of women with personal satisfaction. Also, fatigue from prolonged hospitalization caused 56% of men and 44% of women to be discharged. For hospital‐related reasons, being educational was the most important factor at 30%. Regarding the reasons related to the healthcare providers, lack of satisfaction with the doctor's services was the most common reason (51.6%). Prolonged hospitalization and improper care were the reasons for discharge from both educational and social security hospitals. Dissatisfaction with being educated was only related to university hospitals, where students mostly care for patients. This would prolong the care period, lead to improper care by the specialist, and increase the costs of the patients.

In the study of Asadi et al.,^21^ reasons related to the patient (a feeling of recovery [62.8%], dissatisfaction with medical and nursing services [85%]) and the hospital (failure of diagnostic and therapeutic equipment [94%]) were the most common factors of discharge with personal satisfaction.^21^ In Motazaedi et al.,^18^ patient‐related reasons, prolongation of hospitalization (26.73%), as well as care issues suggested by hospital staff (24.6%) and doctors (19.84%) were the most common factors of discharge with personal satisfaction. Also, the educational nature of the hospital (7.14%), dissatisfaction with medical equipment (27.32%), and high costs (21.51%) were the main factors for discharge with personal satisfaction.^18^ In Abuzeyad et al.'s^22^ study, the main reason for discharge with personal satisfaction was the refusal of surgery and prolonged hospitalization. In university hospitals, by prolonging the hospitalization period and not performing care, dissatisfaction was created. Based on these conditions, doctors advised patients to be discharged with personal consent and go to a private hospital for follow‐up care.

In this study, 95% of patients had basic insurance, and 88.4% did not have supplementary insurance. Therefore, there was no significant relationship between discharge with personal satisfaction and insurance (*p* = 0.28). This condition caused patients hospitalized in social security hospitals, where most insured are social security, and university hospital patients with social security or health insurance, to have basic insurance. There was no relationship between insurance conditions and discharge with personal consent.

In other studies, it has been stated that most patients had basic insurance coverage.^16^, ^21^ Najiba Rasool showed a significant statistical relationship between discharge by personal consent and having basic and supplementary insurance; people with basic insurance and those without supplementary insurance were more motivated to be discharged by personal consent.^16^ Kamaldini et al.^20^ stated that most patients had basic health insurance; they found no statistically significant relationship between discharge with personal satisfaction and having insurance.

Financial problems were the cause of 13.3% of discharges with personal satisfaction. In this study, the average length of stay of people who consented since they had financial problems was 9.91%, 3.75% felt well, and 2.3% were not native. Prolonged hospitalization for people with financial problems caused their expenses to increase; on the other hand, they had to leave the hospital with their consent to earn money for the family. There was a significant relationship between discharge with personal satisfaction and length of hospitalization (*p* < 0.001), patient's problems, and the type of hospital (*p* = 0.04).

## 5 CONCLUSION

The most common reason for discharge with personal satisfaction was related to the patient (the feeling of recovery 47.2%), the hospital (being educational 30%), and the staff (dissatisfaction with the doctor's services 51.3%). Therefore, the feeling of recovery before the completion of the care period makes the patient think he/she is better and can be discharged. Also, in teaching hospitals, patients are dissatisfied due to the treatment of patients by medical students and less presence of specialists. In addition, the advice of specialists to refer patients to private hospitals to receive more wages causes dissatisfaction with the doctor's services. Therefore, paying more attention to monitoring the provision of services, establishing proper communication between the patient and the doctor, and increasing the awareness of the patient and parents about the possible complications of discharge against medical recommendations can help reduce the discharge with personal satisfaction.

## 6 STUDY LIMITATION

Hospital information systems have a section to record the reason for discharge with personal consent by nurses. Unfortunately, the discharge with personal consent was recorded, but the main reason was not. In this study, to find the main reason for discharge with personal consent, patients or their companions were contacted. Some were not available, did not answer, or had forgotten the main reason, which was one of the limitations of the study.

## AUTHOR CONTRIBUTIONS

**Zahra Babaei**: Conceptualization; Data curation. **Masoumeh Alizadeh**: Conceptualization; Data curation. **Soodeh Shahsawari**: Formal analysis; Methodology; Validation; Visualization. **Azar Jihoni‐Kalhori**: Data curation; Software; Validation; Visualization. **Ramin Cheraghbeigi**: Supervision; Validation; Visualization; Writing—original draft. **Rahman Sotoudeh**: Data curation; Investigation; Resources. **Ali Mohammadi**: Conceptualization; Data curation; Funding acquisition; Investigation; Methodology; Project administration; Resources; Software; Writing—original draft; Writing—review & editing.

## CONFLICT OF INTEREST STATEMENT

The authors declare no conflicts of interest.

## TRANSPARENCY STATEMENT

The lead author Ali Mohammadi affirms that this manuscript is an honest, accurate, and transparent account of the study being reported; that no important aspects of the study have been omitted; and that any discrepancies from the study as planned (and, if relevant, registered) have been explained.

## Data Availability

The data supporting this study's findings are available on request from the corresponding author. The data are not publicly available due to privacy or ethical restrictions.
